# Assessment of Objectively Measured Physical Activity Levels in Individuals with Intellectual Disabilities with and without Down's Syndrome

**DOI:** 10.1371/journal.pone.0028618

**Published:** 2011-12-21

**Authors:** Alexander C. Phillips, Anthony J. Holland

**Affiliations:** Department of Psychiatry, University of Cambridge, Cambridge, United Kingdom; Karolinska Institutet, Sweden

## Abstract

**Objective:**

To investigate, using accelerometers, the levels of physical activity being undertaken by individuals with intellectual disabilities with and without Down's syndrome.

**Methods:**

One hundred and fifty two individuals with intellectual disabilities aged 12–70 years from East and South-East England. Physical activity levels in counts per minute (counts/min), steps per day (steps/day), and minutes of sedentary, light, moderate, vigorous, and moderate to vigorous physical activity (MVPA) measured with a uni-axial accelerometer (Actigraph GT1M) for seven days.

**Results:**

No individuals with intellectual disabilities met current physical activity recommendations. Males were more active than females. There was a trend for physical activity to decline and sedentary behaviour to increase with age, and for those with more severe levels of intellectual disability to be more sedentary and less physically active, however any relationship was not significant when adjusted for confounding variables. Participants with Down's syndrome engaged in significantly less physical activity than those with intellectual disabilities without Down's syndrome and levels of activity declined significantly with age.

**Conclusions:**

Individuals with intellectual disabilities, especially those with Down's syndrome may be at risk of developing diseases associated with physical inactivity. There is a need for well-designed, accessible, preventive health promotion strategies and interventions designed to raise the levels of physical activity for individuals with intellectual disabilities. We propose that there are physiological reasons why individuals with Down's syndrome have particularly low levels of physical activity that also decline markedly with age.

## Introduction

When compared with the general population individuals with intellectual disabilities (also referred to as learning disabilities) experience significantly higher rates of morbidity [1], mortality [2], and health inequalities than the general population [3], [4]. The term ‘intellectual disabilities’ refers to a very heterogeneous group of individuals with varied needs and the reasons for these health inequalities are likely to be multiple but low levels of physical fitness [5] and high rates of obesity are contributing factors [6]. For the majority of the population the health benefits of regular physical activity are well established [7]–[9], however, individuals with intellectual disabilities have not been included in the major epidemiological physical activity studies [10], [11] and recent government policy [12], [13] and initiatives [14] have not adequately promoted physical activity for this group. Given their possible dependency on others and in the context of the social circumstances where individuals with intellectual disabilities live, physical activity may be a low priority.

To date, there is only a small literature on the physical activity levels of children [15] and of adults [16] with intellectual disabilities, with the data collected being limited due to the fact that mostly self or proxy reports, or pedometers were used. The former methods have well-known methodological weaknesses [17] and, although the latter are better, they do not give information about whether wearers are meeting physical activity recommendations. Accelerometers are more sophisticated motion sensors compared to pedometers [18]. There have been a small number of studies using this methodology in individuals with intellectual disabilities on small sample sizes (<50), and mostly including relatively young participants with mild to moderate intellectual disabilities with few mobility difficulties, or purposively selected participants based on a particular diagnoses (e.g., Down's syndrome, those with obesity) [19]–[26]. Given these limitations the aims of this study were to investigate, using accelerometers, the levels of physical activity undertaken by a large sample of individuals with intellectual disabilities, to estimate the percentage of participants meeting physical activity recommendations, and to study any association with age, gender, and level of intellectual disabilities. Due to indications from previous research that individuals with Down's syndrome have particularly low levels of physical fitness [5] and high levels obesity [6] we have also compared those with Down's syndrome and those with intellectual disability without Down's syndrome.

## Methods

### Ethical approval and consent procedures

Ethical approval for the study was granted by the Cambridgeshire 3 Research Ethics Committee in April 2008. The research procedures of the Mental Capacity Act 2005 were adhered to assess participant's capacity to consent to take part in the study. For children (12–15 years), the written consent of their parents was obtained. For adults (>16 years), written consent was obtained from those participants judged to have the capacity to consent. Those participants who were judged to lack the capacity to consent, written agreement for the individual to participate was obtained from either a personal or nominated consultee. Only five adults were not able to provide consent themselves. In each case their mother or father supported their participation in the study. Data were collected between July 2008 and May 2010.

### Recruitment

Participants were recruited from the East and South East of England through local care providers, charities, schools, colleges, and clubs. These organisations were asked to identify possible participants based on the selection criteria that participants were known to intellectual disability services, were aged 12 years and above, and could walk unaided (i.e., without a wheel chair or walking aid). Possible participants and their main carer (relative or paid) were sent or given a study information pack and asked to contact the first author (ACP) if they were interested in participating. Unfortunately it was not possible to keep accurate records of how many participants were approached to take part in the study. If participation was agreed then a date was set for the researcher to visit the participant's home residence at a time that was convenient for them. During the home visit several measurements were taken.

### Level of intellectual disability

Level of intellectual disability was assessed using the Leicestershire Intellectual Disability tool which is a measure that combines seven questions on writing, dressing, speech, preparing food, feeding, empathy, and community use [27]. Level of intellectual disability is determined by the total score of the tool using the ICD-10 criteria for mild, moderate, severe, and profound intellectual disability. The Leicestershire Intellectual Disability tool has a diagnostic accuracy of 91% as compared to the Vineland Adaptive Behavior Scale [28]. The measure was administered by the researcher to an individual who knew the participant well (e.g., parent or carer).

### Measurements of height, weight, body mass index and percentage body fat

Weight and height were measured according to set procedures [29]. Weight was measured to the nearest 0.1 kg with the participant dressed in lightweight weight clothing and with no shoes using a calibrated electronic scale (Seca 813, Seca Ltd, Birmingham, UK). Height was measured by the stretch stature method to the nearest 0.5 cm using a portable stadiometer (Seca Leicester Height Measure, Invicta Plastics, Leicester, UK). Body mass index (BMI) was transferred into age and gender appropriate cut-offs for underweight [30], normal weight, overweight, or obese [31], [32].

### Assessment of physical activity

Physical activity was assessed using the Actigraph GT1M accelerometer (Actigraph LLC, Pensacola, FL, USA) for seven consecutive days. The accelerometer was distributed to the participant during the home visit and returned by post to the study office. The accelerometer was fitted to an elastic waistband and attached to the participant's right hip. Instructions were given to the participant and carer both verbally and in writing on how to wear the accelerometer during all waking hours except while bathing, showering, swimming, and playing contact sports. Additionally, participants and carers were asked to record the time the accelerometer was put on and taken off each day and the reason for doing so using a time sheet (e.g., to go bed, swimming, or for contact sports, or for any periods of cycling). The time sheet data was not used in the analysis, since subjective interpretation of time sheet data is required. However, observations from the time sheets and observed counts indicate that the degree of underestimation of overall physical activity was minimal.

### Data analysis

Data from the accelerometer were downloaded onto a computer upon return and processed using a custom written program (MAHUffe, available at www.mrc-epid.cam.ac.uk). Data from the first seven days of complete recordings were used. If participants missed a day of recording due to unforeseen circumstances they were instructed to wear the accelerometer for an extra day. For inclusion in the analysis each participant needed a minimum wear time of 10 hours per day. Non-wear time was defined as sequences of 10 or more consecutive minutes of zero counts. In case of monitor failure or lack of data, participants were asked to re-wear the monitor for a further week.

Accelerometers were programmed to measure activity in 5 second epochs. Outcome variables were total physical activity (counts/min), steps per day (steps/day), and time spent (mins/day) in sedentary, light, moderate, vigorous, and moderate to vigorous physical activity (MVPA) intensities. Published activity intensity cutpoints for children (12–15 years) and adults (>16 years), which had been used in previous studies, were used to estimate the time spent in different activity intensities [10], [11]. Time spent in MVPA was defined as >2802 counts/min for children and >2020 for adults.

Time spent in MVPA and bouts of 10 or more minutes of MVPA were used to estimate the percentage of children and adults with intellectual disabilities meeting physical activity recommendations established by the UK Chief Medical Officers [8]. Briefly children and young people (5–18 years) should accumulate 60 or more minutes of MVPA seven days a week, and adults (>19 years) should accumulate 150 or more minutes of MVPA a week in bouts of at least 10 minutes.

All data analysis was performed using SPSS 15.0 for Windows (Statistical Package for the Social Sciences Inc, Chicago, IL), and significance set at *p*<.05. Each variable was checked for missing data and outliers. Outliers, defined as more than three times the inter-quartile range from the upper quartile boundary, were excluded. Descriptive statistics were calculated for all variables and assessed for normality and homogeneity of variance.

Initial analyses were performed by independent *t*-tests and analysis of variance (ANOVA) to identify differences that could confound comparisons (age, gender, level of intellectual disability, residential setting, and presence of Down's syndrome) between groups. Analysis of covariance (ANCOVA) adjusting for confounding variables as necessary was performed to detect group differences. Partial correlations adjusted for confounding variables were performed to examine the relationship between age and physical activity variables.

## Results

### Demographics and descriptive data

An initial sample of 171 participants known to services for individuals with intellectual disabilities was recruited. Nineteen participants were eliminated from the final analysis because of not completing all testing, mostly due to a failure to wear the accelerometer for the required time period. The final sample was 152 participants aged between 12 and 70 years with mild to severe levels of intellectual disabilities including 61 with idiopathic intellectual disabilities, 79 with Down's syndrome, nine with autism spectrum conditions, 1 with Russell-Silver syndrome, 1 with Treacher Collins syndrome, and 1 with Beckwith-Wiedemann syndrome. Many participants had one or more health problems that were, when necessary, controlled with medication. Table 1 presents the main descriptive characteristics for males and females who were included in the analyses. Independent samples t-tests revealed statistically significant differences in height [*t*(150) = 8.9, *p*<.001] and BMI [*t*(150) = −3.7, *p*<.001] between male and female participants.

**Table 1:** Descriptive data for male and female participants.

Category	Subcategory	Males (*n* = 74)	Females (*n* = 78)
**Age** (years)		33.6 (14.7)	
**Age range**	12–15 years (%)	4.1	5.1
	16–34 years (%)	50.0	48.7
	35–44 years (%)	23.0	17.9
	45–54 years (%)	12.2	11.5
	55–64 years (%)	10.9	16.7
**Presence of Down's syndrome** (%)		44.6	59.0
**Height** (cm)		164.7 (11.1)	149.9 (9.4)*
**Weight** (kg)		71.9 (16.9)	68.2 (16.0)
**BMI** (kg/m^2^)		26.5 (6.3)	30.3 (6.2)*
**BMI category**	Underweight (%)	2.7	1.3
	Normal range (%)	34.2	25.3
	Overweight (%)	37.0	29.1
	Obese (%)	26.0	44.3
**Level of intellectual disability**	Mild (%)	33.8	38.0
	Moderate (%)	39.4	33.8
	Severe (%)	26.8	28.2
**Residential setting**	With parents (%)	43.2	37.2
	Care home (%)	39.2	50.0
	Supported living (%)	17.6	12.8
**Employment/daytime placement**	Student (%)	27.0	26.9
	Day centre (%)	14.9	26.9
	Social enterprises (%)	16.2	12.8
	Day centre and social enterprises (%)	21.6	19.2
	Part/full-time work (%)	10.8	7.7
	None (%)	9.6	6.4

Data are mean and (standard deviation) unless stated otherwise.

*statistically significant difference between males and females (*p*<.05).

### Main physical activity data

Table 2 presents the main physical activity data for male and female participants by age group. The total sample averaged 6334 steps per day over the seven days ranging from a low of 743 to a high of 18,191 steps per day. Regardless of age or gender, on average, most of the waking day was spent being sedentary (608.1 mins/day) followed by light (120.7 mins/day) and then moderate physical activity (33.7 mins/day), with very little time engaged in vigorous physical activity (2.1 mins/day). Most MVPA for both adult males (*M* = 1.0, *SD* = 2.3) and females (*M* = 0.4, *SD* = 1.6) was accrued through short bouts (<10 minutes) and, therefore, does not count towards meeting government physical activity recommendations. No adolescent or adult participants met current UK physical activity recommendations [8].

**Table 2:** Physical activity data by gender and age group.

MEN					
Variable	12–15 years (*n* = 3)	16–34 years (*n* = 37)	35–44 years (*n* = 17)	45–54 years (*n* = 9)	55+ years (*n* = 8)
Total physical activity (counts/min)	836.2 (619.0)	694.7 (184.2)	739.0 (418.3)	602.0 (182.7)	585.0 (264.8)
Sedentary (mins/day)	558.3 (61.1)	604.0 (65.2)	586.7 (143.8)	603.6 (96.7)	648.2 (95.1)
Light (mins/day)	151.9 (19.1)	112.9 (30.5)	121.4 (41.5)	131.3 (36.2)	119.9 (52.6)
Moderate (mins/day)	19.8 (4.5)	39.6 (17.3)	42.8 (33.0)	29.5 (18.6)	38.3 (33.9)
Vigorous (mins/day)	8.5 (10.5)	2.6 (2.8)	2.3 (3.2)	1.8 (2.7)	0.7 (0.5)
MVPA_all_ (mins/day)	28.2 (14.9)	42.2 (19.1)	45.2 (34.1)	31.3 (20.1)	39.0 (34.2)
MVPA_+10_	-	0.9 (1.9)	1.6 (3.2)	0.0 (0.0)	1.8 (3.4)
Steps (steps/day)	7181 (179)	6558 (2493)	7376 (4199)	6682 (2831)	7723 (5168)

WOMEN					
Variable	12–15 years (*n* = 4)	16–34 years (*n* = 38)	35–44 years (*n* = 14)	45–54 years (*n* = 9)	55+ years (*n* = 13)
Total physical activity (counts/min)	680.0 (91.9)	577.0 (138.7)	556.3 (87.7)	577.7 (485.0)	485.0 (170.7)
Sedentary (mins/day)	609.3 (80.6)	644.2 (40.6)	560.4 (84.7)	576.0 (49.5)	605.1 (86.0)
Light (mins/day)	144.2 (49.2)	113.6 (27.8)	139.9 (38.2)	143.2 (54.0)	104.9 (33.2)
Moderate (mins/day)	23.0 (7.5)	30.0 (12.3)	30.8 (9.9)	33.5 (15.3)	20.7 (14.8)
Vigorous (mins/day)	3.9 (1.1)	2.1 (3.3)	1.0 (0.4)	2.0 (2.1)	0.8 (0.4)
MVPA_all_ (mins/day)	26.9 (6.5)	32.1 (13.5)	31.8 (9.9)	35.4 (16.5)	21.5 (14.7)
MVPA_+10_	-	0.4 (1.3)	0.8 (2.9)	0.0 (0.0)	0.0 (0.0)
Steps (steps/day)	6918 (749)	5648 (1831)	6274 (2021)	6751 (2090)	4649 (2126)

Data are displayed as mean and (standard deviation).

### Physical activity differences by age and gender

Physical activity across age bands are shown in Table 2 with a trend towards an increase in sedentary behaviour and a decrease in physical activity in later life, however, any relationship with age was not significant. Differences in physical activity by gender are shown in Table 3. ANCOVA adjusted for age, presence of Down's syndrome, level of intellectual disability, and residential setting revealed males engaged in significantly more total physical activity [*F*(1, 146) = 8.3, *p* = .005], MVPA [*F*(1, 146) = 7.9, *p* = .006], and steps per day [*F*(1, 146) = 6.2, *p* = .014] than females.

**Table 3:** Physical activity data for male and female participants.

Variable	Males (*n* = 74)	Females (*n* = 78)
Total physical activty (counts/min)	665.0 (224.2)	564.1 (146.3)*
Sedentary (mins/day)	605.3 (95.3)	616.0 (70.6)
MVPA (mins/day)	40.4 (24.1)	30.2 (13.7)*
Steps (steps/day)	6978 (3269)	5741 (1918)*
Wear time (mins/day)	767.4 (83.5)	768.5 (66.0)

Data are mean and (standard deviation).

Analysis adjusted for age, presence of Down's syndrome, level of intellectual disability, and residential setting.

*statistically significant difference between male and female participants (*p*<.05).

### Physical activity differences by level of intellectual disability

Physical activity data by level of intellectual disability are shown in Table 4. There was a general trend towards those with more severe levels of intellectual disability being more sedentary and less physically active, however, ANCOVA adjusted for confounding variables only found a significant trend towards a decreasing number of steps per day as the level of intellectual disability becomes more severe [*F*(2, 145) = 6.2, *p* = .003].

**Table 4:** Level of intellectual disability physical activity data.

Variable	Mild (*n* = 54)	Moderate (*n* = 56)	Severe (*n* = 42)
Total physical activty (counts/min)	663.0 (161.9)	615.0 (225.6)	550.6 (175.2)
Sedentary (mins/day)	607.7 (87.6)	604.9 (84.7)	630.0 (60.0)
MVPA (mins/day)	40.1 (19.0)	33.6 (17.5)	28.7 (17.0)
Steps (steps/day)	7323 (2461)	5925 (2379)	5400 (2527)*
Wear time (mins/day)	779.7 (71.8)	765.0 (75.1)	775.0 (65.0)

Data are displayed as mean and (standard deviation).

Analysis adjusted for gender, age, presence of Down's syndrome, and residential setting.

*statistically significant difference between level of intellectual disability (*p*<.05).

### Physical activity and Down's syndrome

Physical activity data for participants with and without Down's syndrome are shown in Table 5. ANCOVA adjusted for confounding variables found participants with intellectual disabilities without Down's syndrome engaged in significantly more total physical activity [*F*(1, 146) = 10.3, *p*<.001], MVPA [*F*(1, 146) = 11.5, *p*<.001], steps per day [*F*(1, 146) = 12.4, *p*<.001], and were significantly less sedentary [*F*(1, 146) = 5.0, *p* = .027] than participants with Down's syndrome.

**Table 5:** Physical activity data for participants with and without Down's syndrome.

Variable	ID without DS (*n* = 73)	DS (*n* = 79)
Total physical activty (counts/min)	687.0 (260.8)	570.1 (191.0)*
Sedentary (mins/day)	590.8 (81.0)	627.9 (82.7)*
MVPA (mins/day)	41.6 (23.0)	29.8 (15.6)*
Steps (steps/day)	7301 (3053)	5541 (2214)*
Wear time (mins/day)	751.6 (73.4)	763.0 (73.3)

Data are mean and (standard deviation).

Analysis adjusted for gender, age, level of intellectual disability, and residential setting.

*statistically significant difference between participants with and without DS (*p*<.05).

Age was more strongly associated with a decrease in total physical activity (*F*(1, 146) = 8.9, *p*<.001) and MVPA (*F*(1, 146) = 7.5, *p* = .001) in those with Down's syndrome than those with intellectual disabilities without Down's syndrome adjusting for gender, level of intellectual disability, and residential setting. This suggested that age was having a more dramatic effect in those with Down's syndrome. This difference is illustrated by the fact that in contrast to those with intellectual disabilities without Down's syndrome, partial correlations adjusted for gender, level of intellectual disability, and residential setting found a statistically significant decrease with age in total physical activity [*r*(74) = −.30, *p* = .010] and MVPA [*r*(74) = −.27, *p* = .019] only for those with Down's syndrome.

## Discussion

This study provides the largest published data set on objectively measured physical activity in individuals with intellectual disabilities. The procedures used in this study are consistent with standard accelerometry practices used by researchers in the UK [11] and in other countries [10], and thereby provide valuable information about the physical activity levels of individuals with intellectual disabilities in England.

### Principles findings of the study

Worryingly, no children or adults in the current study met current [8] or former [7] physical activity recommendations. This is lower than the 3.5% of children and 5% of adults in the general population meeting such recommendations with the exception that children and adults were engaged in slightly more MVPA per day than data presented on the general population in the 2008 Health Survey for England [11]. However, children and adults with intellectual disabilities in the current study engaged in more sedentary behaviour than children and adults in the 2008 Health Survey for England. MVPA was very sporadic with few participants managing sustained bouts which is consistent with previous research on individuals with intellectual disabilities [22]. Vigorous physical activity appeared to be non-existent in participants in this study, which is also consistent with previous research on individuals with intellectual disabilities [20], [33], [34].

In agreement with research from the general population [10], [11], [35], but in contrast with previous research involving individuals with intellectual disabilities [36], [37], males were significantly more physically active than females. This difference in findings with previous intellectual disability research may be explained by methodological differences (e.g., questionnaire, interview, direct observation). Again, in agreement with previous studies from the general population [10], [11] and from studies involving individuals with intellectual disabilities [36], [37], physical activity decreased with age, however, for the group as a whole this was not significant after adjustment for confounding variables. This study found physical activity decreased with the level of intellectual disability, which is not surprising as it would be expected that individuals with higher ability have, in general, fewer restrictions, less need for staff supervision, and more independence to be physically active [38]. However, after adjusting for potential confounders, a significant decrease was only found in steps per day.

The striking findings of the lower physical activity levels and a marked reduction with age in individuals with Down's syndrome are extremely important. These results shed light on possible reasons why this group has consistently been found to have poorer physical fitness [5] and higher levels of obesity [6] compared to those with intellectual disabilities without Down's syndrome. This would suggest that unique age-related physiological factors maybe at play, which reduce the ability of individuals with Down's syndrome to engage in physical activity. Down's syndrome is associated with conditions that contribute to low levels of physical activity and fitness compared to those with intellectual disabilities without Down's syndrome, e.g., chronotropic incompetence, impaired autonomic function, low muscular strength, and muscle hypotonia [5], [39]. We propose that a possible candidate for the above and underlying age-related pathophysiology may be that of mitochondrial dysfunction resulting in energy deficiency and oxidative stress [40], [41]. Previous research has found mitochondrial dysfunction in Down's syndrome brain tissue, blood cells, fibroblasts, heart [41], and preliminary work carried out by this research group have found in vivo skeletal muscle mitochondrial dysfunction in adults with Down's syndrome.

### Comparison with other studies

There have only been two studies to have used the Actigraph to assess physical activity in individuals with intellectual disabilities. Frey [22] assessed 22 adults with intellectual disabilities from the USA using the Actigraph 7164 and found 28% met US physical activity recommendations [42]. Participants engaged in less MVPA (20 mins/day) but more total physical activity (602 counts/min) than this study. Caution is needed when making comparison between the two studies as the studies differ in the Actigraph model used [43], longer epoch length (one minute) [44], sample size, and the inclusion only of participants with mild intellectual disabilities in the previous study. Recently, Melville [26] presented data from a multi-component weight-loss intervention for 45 adults with intellectual disabilities and obesity from Scotland. Baseline physical activity measurement using the Actigraph GT1M found participants engaged in a similar amount of sedentary behaviour(608 mins/day) to this study but far less light physical activity (70 mins/day) and MVPA (13 mins/day). This difference in physical activity levels may be due to the fact that participants in the Melville study were selected on the basis of a BMI of greater than 30 kg/m^2^.

### Limitations of the study

This study has several limitations which may affect the application of these findings. Physical activity may have been underestimated as the use of a single, waist mounted, uni-axial accelerometer will not measure physical activity during upper-body and non-weight bearing activities (e.g., stereotypy, load carrying, swimming, cycling). However, few participants reported significant amounts of these activities. It is also possible that there may be differences in metabolic equivalent derived cutpoints between individuals with and without intellectual disabilities. To the authors knowledge there has been no published work exploring this issue. However, the use of Actigraph raw scores (counts/min), which are deemed by many researchers to be a more appropriate way of analysing physical activity in a field setting [45], did not change any of the results with respect to age, gender, level of intellectual disability, and Down's syndrome.

Even though all attempts were made to obtain a representative sample, there is the possibility that the participants who volunteered for this study are more active than their average peers with intellectual disabilities, and although the sample size was large for studies on individuals with intellectual disabilities, the sample size for adolescents was small due to difficulties in recruiting this group. Future studies should replicate this study using a larger sample size of adolescents with intellectual disabilities. The cross-sectional nature of results must be considered when interpreting the results of this study. Future studies with a longitudinal design, assessing changes in physical activity over time will be valuable in exploring causal relationships.

The effect of medications on physical activity was not evaluated. Further research is needed to understand the impact of medications on physical activity levels in this population as individuals with intellectual disabilities are prescribed higher amounts of medication compared to the general population [46]. Physical activity is known to show seasonal variation [47] and such variation was not investigated for this study. Examination of seasonal variation in physical activity among individuals with intellectual disabilities may help identify individual and environmental factors that may be targets of tailored strategies to enhance participation.

### Conclusions and clinical implications

In recent decades, individuals with intellectual disabilities have experienced many advances in education, work, living arrangements, and human rights [12], [13]. Despite these developments, they experience significant health inequalities [3], [4] with increased rates of morbidity [1] and mortality [2]. Worryingly, in the current study there were no children or adults that achieved the recommended amount of physical activity for health. Individuals who do not engage in the recommended levels of physical activity present higher rates of mortality and higher incidence rates of many chronic diseases [7]–[9]. The findings of this study suggest that individuals with intellectual disabilities, especially those with Down's syndrome, may be at risk of developing diseases associated with physical inactivity.

The major epidemiological studies [10], [11] and health promotion strategies [14] that have been completed over the last ten years demonstrating the enormous benefit to health derived from physical activity have not included individuals with intellectual disabilities in their designs. This is a group who may be dependent on others for support and whose activities and lifestyle are significantly under the influence of others [48]. There is a need for well-designed, accessible, preventive health promotion strategies and interventions designed to raise the levels of physical activity for individuals with intellectual disabilities which take into account the unique barriers to physical activity this group faces [49], [50].
